# Systematic Review and Meta-Analysis of the Clinical Efficacy and Adverse Effects of Chinese Herbal Decoction for the Treatment of Gout

**DOI:** 10.1371/journal.pone.0085008

**Published:** 2014-01-21

**Authors:** Liang Zhou, Ling Liu, Xiaoyu Liu, Pinyi Chen, Ling Liu, Yanqi Zhang, Yazhou Wu, Julia Christine Pettigrew, Dixiang Cheng, Dong Yi

**Affiliations:** 1 Department of Health Statistics, College of Preventive Medicine, Third Military Medical University, Chongqing, China; 2 P. E. Department of Physical Education of Chongqing University of Arts and Sciences, Chongqing, China; 3 University of Washington, School of Arts and Sciences, Department of Biological Sciences and Department of Asian Language and Literature, Seattle, Washington, United States of America; 4 School of Software Engineering, Chongqing University of Posts and Telecommunications, Chongqing, China; University of California San Francisco, United States of America

## Abstract

**Background:**

In East Asia, numerous reports describe the utilization of traditional Chinese herbal decoctions to treat gout. However, the reported clinical effects vary.

**Objectives:**

In this study, we reviewed and analyzed a large number of randomized controlled clinical trials to systematically assess the clinical efficacy and adverse reactions of Chinese herbal decoctions for treating gout.

**Methods:**

We performed a comprehensive search of databases, such as PubMed, EMBASE, the Cochrane Central Register of Controlled Trials, Chinese biomedical literature database, et al. In addition, we manually searched the relevant meeting information in the library of the Third Military Medical University.

**Results:**

Finally, 17 randomized controlled trials with a sample size of 1,402 cases met the criteria and were included in the study. The results of the meta-analysis showed that when gout had progressed to the stage of acute arthritis, there was no significant difference in clinical efficacy between Chinese herbal decoctions and traditional Western medicine, as indicated based on the following parameters: serum uric acid (standardized mean difference (SMD):0.35, 95% confidence interval (CI): 0.03 to 0.67), C reactive protein (SMD: 0.25, 95% CI: −0.18 to 0.69), erythrocyte sedimentation rate (SMD: 0.21, 95% CI: −0.02 to 0.45) and overall clinical response (relative risk (RR): 1.05, 95% CI: 1.01 to 1.10). However, the Chinese herbal decoction was significantly better than traditional Western medicine in controlling adverse drug reactions (RR: 0.06, 95% CI: 0.03 to 0.13).

**Conclusions:**

Through a systematic review of the clinical efficacy and safety of Chinese herbal decoctions and traditional Western medicine for the treatment of gout, we found that Chinese herbal decoction and traditional Western medicine led to similar clinical efficacy, but the Chinese herbal decoctions were superior to Western medicine in terms of controlling adverse drug reactions.

## Introduction

Gout, occurs when purine metabolism is disrupted, which causes increased synthesis or reduced discharge of uric acid and subsequently results in the excruciatingly painful inflammatory Arthritis [1]. When the serum uric acid concentration is too high, uric acid is deposited in the joints, cartilage and kidney in the form of a sodium salt, leading to a type of arthritis in which the tissue initiates an inflammatory response against foreign bodies. The clinical manifestations of gout include the characteristic recurrent acute arthritis, tophi deposition, chronic gouty arthritis and deformed joints [2], [3]. In addition, this disease often affects the kidneys, causing chronic interstitial nephritis and the formation of urinary tract stones composed of uric acid. In severe cases, gout can lead to damaged or maimed joints and renal insufficiency [4].

In China, gout is more commonly found in the middle-aged and the elderly population, this is, the population over the age of 40. However, improved economic conditions have led to dietary changes. The high incidence of gout is no longer restricted to the elderly, and gout has shown a trend of early onset in younger populations [5]. In addition, a British population-based study reported that the prevalence of gout was 1.4% in the UK and Germany during the year 2000–2005. [6]. During the past 20 years, the prevalence of gout has increased in European countries and the United States. The UK gout prevalence increased from 1.19% in 1990 to 1.4% in 1999, and the incidence of gout and hyperuricemia in the United States showed a similar upward trend from 1990 to 1999 [7]. Compared with Europe and the United States, the prevalence of gout in Asia began increasing after World War II and has shown a significantly increasing trend over the past 20 years. The hyperuricemia prevalence in Japanese male adolescents increased from 3.5% in 1991 to 4.5% in 1992 [8]. In the city of Qingdao in China from 2002–2004, the gout prevalence increased from 3.6/1000 to 5.3/1000. [9], [10].

The conventional Western medicine treatment for the disease focuses on the treatment of the primary disease, that is, high uric acid. Colchicine and non-steroid anti-inflammatory drugs are mainly utilized to treat the disease during the acute phase, and glucocorticoids can also be adopted for the therapy. However, the above treatments are likely to result in a gastrointestinal tract reaction, tissue and organ damage and other adverse reactions [11], [12], which hamper efforts to prolong the treatment [13].

A decoction of Chinese herbs refers to the liquid formulation that is obtained by boiling pieces or coarse grains of herbs in water or soaking the crude medicine in boiling water followed by filtration [14]. Chinese herbal decoction has a long history of treating gout in China and also has certain unique clinical effects. In the past decade, an increasing number of studies comparing Chinese herbal decoction and traditional Western medicine for the treatment of gout have been reported. However, there are some disparities in the treatment method and efficacy among these studies [15], which affects the reliability and replicability of the conclusions and makes it difficult for the field of medicine to appreciate the findings [16]. Therefore, a rational systematic review is of great significance.

Treatment of gout and other metabolic diseases by traditional Chinese medicine has always been the focus of our team. This study combined the principle of evidence-based medicine and the dialectical theory of traditional Chinese medicine to perform a systematic review/meta-analysis of the clinical efficacy and safety of treatment regimens for the acute arthritis phase of gout, thereby providing guidance and references for the clinical application of traditional Chinese medicine.

## Materials and Methods

### Experimental design

The clinical designs in all reports included in this study were clinical randomized controlled trials (RCTs). The trials were divided into experimental and control groups based on the intervention methods, with the experimental group receiving the Chinese herbal decoction alone and the control group receiving only Western medicine. The time of publication was restricted to the period from January 2001 to June 2012, and the journals' languages were restricted to Chinese and English.

### Subjects

According to the diagnostic criteria created by the American Rheumatism Association in 1977, all subjects included in the study were diagnosed with primary gout in the phase of acute arthritis [17]. Subjects with blood diseases or cancer were excluded. Pregnant women were also excluded.

### Database search strategy

We used “Gout,” “hyperuricemia” and “Pain Paralysis” as the title words, and “Chinese medicine,” “herbal medicine” or “Chinese medicine practice” as the keywords to search the following databases: PubMed, Medline, Chinese Biomedical Literature Database, China Doctoral Dissertations Full-text Database, Chinese Scientific and Technological Journals Database, Traditional Chinese Medicine Database, China Doctoral Dissertations Full-text Database, China Master Dissertations Full-text Database and CENTRAL from the Cochrane Library during the period from January 2001 to June 2012. Additionally, we manually searched for relevant meeting information stored in the library of the Third Military Medical University. The format using for the Medline search was as follows:

#1 gout or podagra

#2 hyperuricemia

#3 Chinese medicine or Chinese herbal medicine or traditional Chinese medicine

#4 #1 and #2 and #3

### Data analysis

Three investigators participated in the data extraction of all publications included in the study. Information, including the first author, publication year, total number of cases included in the experimental group and the control group, intervention methods and endpoint evaluation indicators, was extracted. One investigator (XL) first performed the data extraction, and the second investigator (LL^2^) subsequently re-examined the publication and verified the results. Differences were discussed with the third investigator (PC), and consensus was reached by discussion.

### Endpoint indicators

Enumeration data were divided into 4 categories of cure, markedly effective, effective and ineffective in accordance with the standards of “Guiding Principles for the Clinical Investigation of New Traditional Chinese Medicine”(*cured*, which means arthralgia, arthrocele, the single toe joint swelling pain et al and its associated symptoms have reduced ≥75%, the blood uric acid, erythrocyte sedimentation rate (ESR), C-reactive protein(CRP) have reduced in normal range; *markedly effective*, which means arthralgia, arthrocele, the single toe joint swelling pain etal and its associated symptoms have reduced ≥50%, the blood uric acid, ESR, CRP havereduced; *effective*, which means arthralgia, arthrocele, the single toe joint swelling pain et al and its associated symptoms have reduced ≥30%, he blood uric acid, ESR, CRP have reducedor not; *ineffective*, which means arthralgia, arthrocele, the single toe joint swelling pain et al and its associated symptoms have reduced <30%, he blood uric acid, ESR,CRP haven't reduced.) [18]. The number of patients with effective treatment results in each group was counted based on the categories of cured, markedly effective and effective. Measurement data included three primary indicators: blood uric acid, C-reactive protein and erythrocyte sedimentation rate (ESR).

### Quality assessment of the included articles

The quality of the articles included in this study was assessed using the Cochrane Handbook for Systematic Reviews of Interventions and Jadad scoring [19], [20]. Two reviewers (XL and LL^2^) performed blinded independent evaluation. If inconsistent evaluation results were obtained, a third reviewer (LZ) intervened, and consensus was reached by discussion. The details that were assessed were as follows: 1) whether the test methods were random, 2) whether allocation concealment was achieved, 3) whether blinded tests were adopted and 4) whether patients were lost due to follow-up or quit. The scoring scale was 1–7 (1–3 indicated low quality, and 4–7 indicated high quality).

### Statistical methods

The measurement data were combined using the standardized mean difference (SMD) and 95% confidence interval (CI), and the enumeration data were evaluated using the relative risk (RR) and 95% CI based on the number of patients with effective treatment results in the combined experimental group and control group. The subgroup-meta-analysis and sensitivity analysis were also used in this study. If the heterogeneity across the studies was within the acceptable range (I^2^<50%), a fixed effects model was used to combine the studies. Otherwise, a random effects model was used. RevMan 5.0 software was used to analyze the collected clinical research data.

## Results

### Literature search results

**Figure 1:**
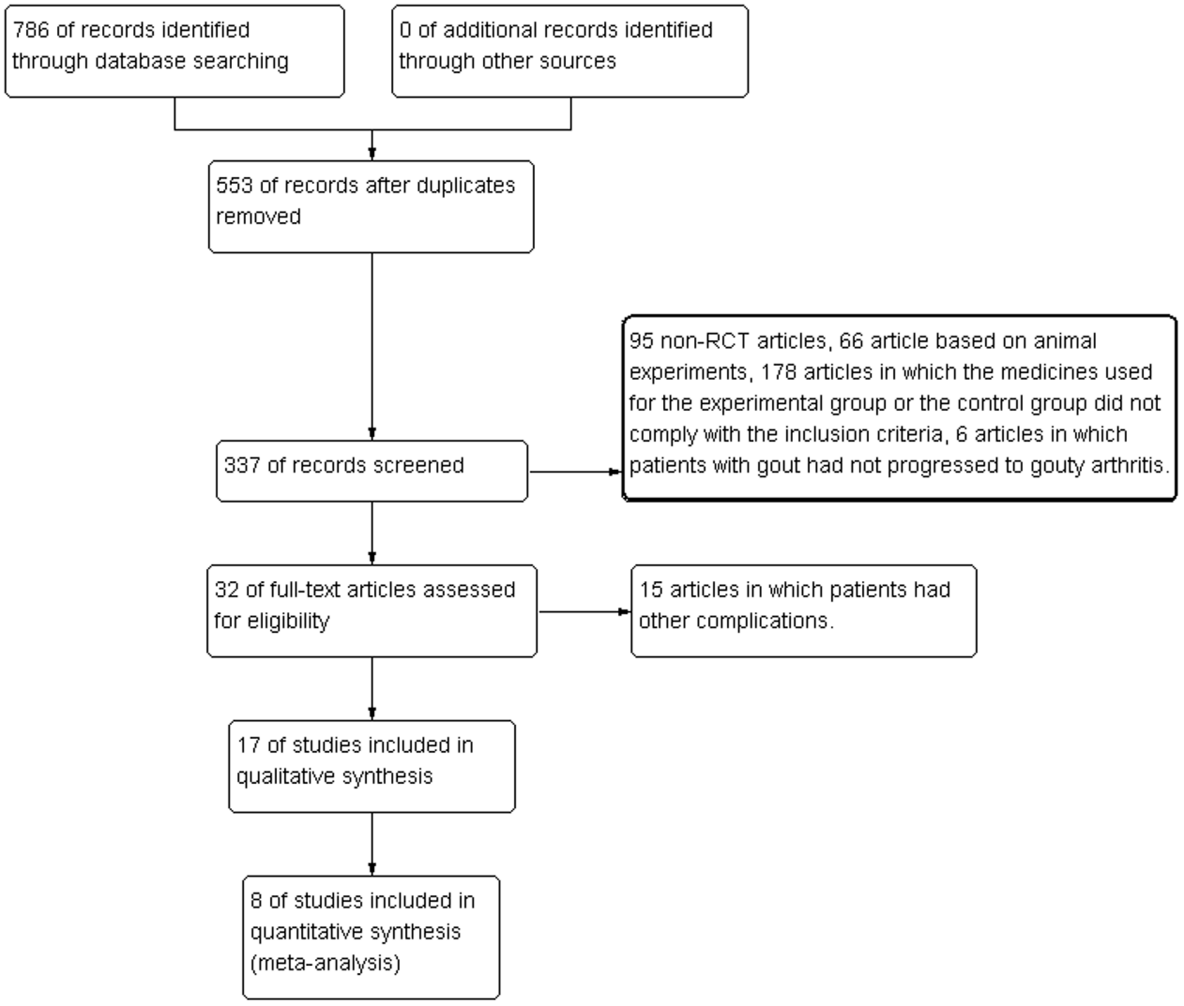
Flowchart of the included articles.

A total of 786 research articles on the treatment of gout using traditional Chinese medicine were identified by searching the electronic databases. Based on the inclusion and exclusion criteria, 377 articles, including duplicated publications, articles with mismatched titles and articles with mismatched subjects, were excluded. The remaining 324 articles were reviewed thoroughly; 95 non-RCT articles, 66 articles based on animal experiments, 178 articles in which the medicines used for the experimental group or the control group did not comply with the inclusion criteria, 6 articles in which patients with gout that had not progressed into gouty arthritis and 15 articles in which patients had other complications were excluded. Thus, 17 studies were included in this study for the systematic review [21], [22], [23], [24], [25], [26], [27], [28], [29], [30], [31], [32], [33], [34], [35], [36], [37] (**Figure 1** **&** **Table 1** **&** **Table 2**).

**Table 1:** Characteristics of the included studies.

Author, Year	Sample size (EG▴)	Sample size (CG▴)	Age (EG)	Age (CG)	Disease severity (EG)	Disease severity (CG)	Intervention methods (/d) (EG)	Intervention methods (/d) (CG)	Duration treatment (/d) (EG/)	Duration treatment (/d) (CG)
Yigong Zhang, 2000 [21] ▪	63	50	20∼76	24∼68	The single joint disease: 54; uratoma:28; kidney damage: 1.	The single joint disease: 39; uratoma:10; kidney damage: 7.	Atractylodes lancea, Poria cocos, Polyporus Umbellatus, Pseudbubus Cremastra Seu pleiones et al.	Colchicine	10	10
Liping Huang, 2003 [22]	32	32	27∼63	27∼63	The course of disease:1∼6 years.	The course of disease:1∼6 years.	Rhizoma Smilacis Glabrae (30 g), Coix Seed (30 g), Pseudbubus Cremastra Seu pleiones (30 g) et al.	Allopurinol (0.1 g*3)	10	10
Pingyong Li, 2004 [23]	60	64	37∼72	34∼72	Primary gout; Kidney, liver damage: 0; coronary heart disease: 0; allergy: 0.	Primary gout; Kidney, liver damage: 0; coronary heart disease: 0; allergy: 0.	Pseudbubus Cremastra Seu pleiones (20 g), Rhizoma Smilacis Glabrae(20 g), Dioscorea tokoro (20 g) et al.	Colchicine (4∼8 mg), Allopurinol (0.3 g), Ibuprofen (0.1 g*3)	10	10
Chongqing Yang, 2006 [24] ▪	30	30	18∼70	18∼70	The course of disease:1∼5 years.	The course of disease:1∼5 years.	Rhizoma Smilacis Glabrae, Dioscorea tokoro Makino, Pseudbubus Cremastra Seu pleiones et al.	Diclofenac Sodium Enteric-coated Tablets (25 mg*3)♦	7	7
Lianqun Qiu, 2007 [25]	34	16	38∼74	35–69	The course of disease:0.5∼20 years	The course of disease:1∼16 years	Rhizoma Smilacis Glabrae (30 g), Semen Brassicae (20 g), Pseudbubus Cremastra Seu pleiones et al.	Allopurinol(0.1 g*2∼3), Meloxicam (7.5 mg)	15	15
Huajie Wang, 2008 [26] ▪	42	41	22∼65	22∼65	Primary gout: 42; kidney, liver damage: 0; coronary heart disease: 0; allergy: 0.	Primary gout: 41; kidney, liver damage: 0; coronary heart disease: 0; allergy: 0.	Rhizoma Smilacis Glabrae, Dioscorea tokoro Makino, Radix Achyranthis Bidentatae, et al.	Colchicine (0.5 mg), Benzbromarone (50 mg)	21	21
Chenglong Tang, 2008 [27]	30	26	28∼68	28∼68	The course of disease: 1 month∼12 years.	The course of disease: 1 month∼12 years.	Atractylodes lancea (10 g), Cortex Phellodendri Chinensis (10 g), Pseudbubus Cremastra Seu pleiones (12 g) et al.	Colchicine (0.5 mg*2) Allopurinol (0.2 g*3)	15	15
Ying Lu, 2008 [28] ▪	120	96	47∼78	47∼78	The single joint disease: 216; uratoma:20; kidney damage: 76; coronary heart disease: 32.§	No independent report based on this data has yet published.	Cortex Phellodendri Chinensis (15 g), Radix Stephaniae Tetrandrae (15 g), Coix Seed (15 g), Radix Angelicae Pubescentis (25 g), Geosaurus, Pseudbubus Cremastra Seu pleiones (25 g) et al.	Colchicine, Allopurinol	30	30
Jin Yang, 2010 [29]	30	30	40∼65	30∼64	The course of disease: 2 ∼10years.	The course of disease: 2 ∼10years.	Cortex Phellodendri Chinensis (10 g), Atractylodes lancea (10 g), Pseudbubus Cremastra Seu pleiones (10 g), et al.	Difene (75 mg), Allopurinol (0.1 g*3)	7	7
Bing Ji, 2010 [30]	30	30	51.77±7.92	52.93±2.87	The course of disease: 5.67±2.87 years.	The course of disease: 5.50±2.76 years.	Rhizoma Smilacis Glabrae (30 g), Rhizoma Polygoni Cuspidati (8 g), Coix Seed (30), Radix Cyathulae (15 g), et al.	Colchicine (0.5 mg*3), Celebrex (0.2 mg)	7	7
Xiaoxia Wang, 2010 [31]	30	30	48.5±15.5	49.1±13.7	The course of disease: 1day∼12 years.	The course of disease: 2 day∼8 years.	Pseudbubus Cremastra Seu pleiones (12 g), Semen Persicae (12 g), Atractylodes lancea (12 g), Rhizoma Alismatis (20 g), et al.	Colchicine (0.5 g*2)	10	10
Yu Ding, 2011 [32]	40	36	28∼73	30∼80	The course of disease: 12 h∼13 years. Recurrence: 12	The course of disease: 10 h∼15 years. Recurrence: 14	Atractylodes lancea (10 g), Cortex Phellodendri Chinensis (6 g), Coix Seed (20 g), Rhizoma Smilacis Glabrae (20 g) et al.	Allopurinol (0.1 g) Colchicine (0.5 g), Indomethacin (25 mg)	7∼21	7∼21
Xizhou Zhang, 2011 [33]	45	45	33∼60	35∼58	The course of disease: 3 month ∼6 years.	The course of disease: 3 month ∼8 years.	Radix Scutellariae (6 g), Coptis chinensis Franch (6 g), Cortex Phellodendri Chinensis (12 g), Lilium brownii (12 g), et al.	Ibuprofen (0.3 g*2), Allopurinol (0.1 g*3)	10	10
Mengyuan Liu, 2011 [34]	30	30	35∼65	39∼65	Kidney, liver damage: 0; coronary heart disease: 0; allergy: 0.	Kidney, liver damage: 0; coronary heart disease: 0; allergy: 0.	Atractylodes lancea (30 g), Cortex Phellodendri Chinensis (10 g), Coix Seed (30 g), Lilium brownii (10 g), et al.	Allopurinol (0.1 g*2) nimesulide (0.1 g)	21	21
Yongkai Huang, 2011 [35]	15	15	50.5±13.3	51.5±14.2	Kidney, liver damage: 0; coronary heart disease: 0; allergy: 0.	Kidney, liver damage: 0; coronary heart disease: 0; allergy: 0.	Rhizoma Smilacis Glabrae (45 g), Dioscorea tokoro Makino (30 g), Coix Seed (30 g), Bombyx Batryticatus (15 g) et al.	Meloxicam Tablets (15 mg)•	7	7
Yadong Han, 2011 [36] ▪	60	48	47∼78	47∼78	The single joint disease: 108; kidney damage: 38; coronary heart disease: 18.§	No independent report based on this data has yet published.	Cortex Phellodendri Chinensis (15 g), Radix Stephaniae Tetrandrae (15 g), Coix Seed (15 g), Lilium brownii (15 g), et al.	Colchicine, Allopurinol	30	30
Baiyan Qin, 2011 [37]	46	46	47.2±11.7	48.0±10.5	The course of disease: 6 h∼23 years. Recurrence: 31	The course of disease: 10 h∼19 years. Recurrence: 29	Fructus Ligustri Lucidi (15 g), Rhizoma Anemarrhenae (10 g), Pseudbubus Cremastra Seu pleiones (15 g) et al.	Colchicine (0.5 mg*2), Allopurinol (0.1 g*3)	10	10

▴ EG Experimental group, CG Control group;

▪ No dose be provided of intervention;

♦ The molecular formula of main ingredients: C14H10Cl2NNa02;

• The molecular formula of main ingredients: C14H13N3O4S2;

§ Including the data of experimental group and control group.

**Table 2:** Outcomes.

Author Year	The overall efficacy 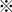 (EG▴)	The overall efficacy 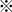 (CG▴)	Blood uric acid concentration (µmol/L) (EG: B/A♦)	Blood uric acid concentration (µmol/L) (CG: B/A♦)	C-reactive protein (mg/L) (EG B/A♦)	C-reactive protein (mg/L) (CG: B/A♦)	ESR (mm/h)▪ (EG: B/A♦)	ESR (mm/h)▪ (CG: B/A♦)	Adverse reactions (EG)	Adverse reactions (CG)
Yigong Zhang, 2000 [21]	0/44/17/2	0/25/22/3	N/A	N/A	N/A	N/A	N/A	N/A	Nausea, diarrhea: 3	Nausea, diarrhea: 42; hematuresis: 4.
Liping Huang, 2003 [22]	0/13/17/2	0/5/19/8	N/A	N/A	N/A	N/A	N/A	N/A	N/A	N/A
Pingyong Li, 2004 [23]	41/0/21/2	30/0/26/4	N/A	N/A	N/A	N/A	N/A	N/A	N/A	N/A
Chongqing Yang, 2006 [24]	7/17/3/3	12/11/5/2	487.27±98.87/404.88±95.48	N/A	N/A	N/A	N/A	N/A	N/A	N/A
Lianqun Qiu, 2007 [25]	11/14/6/3	4/4/5/3	539±42.07/397.41±32.56	528.37±40.28/379.25±30.17	N/A	N/A	73.26±25.17/25.73±10.27	71.39±24.08/26.39±11.07	Nausea, diarrhea: 2	Nausea, diarrhea: 3; Itchy skin:1; liver damaged:3
Huajie Wang, 2008 [26]	0/31/8/3	0/28/9/4	595.43±93.93/326.13±86.26	587.44±103.27/321.96±93.87	N/A	N/A	N/A	N/A	Nausea, diarrhea: 1	Nausea, diarrhea: 3; Itchy skin:1; liver damaged:1.
Chenglong Tang, 2008 [27]	21/7/0/2	18/6/0/2	512.60±35.30/380.40±42.40	523.30±38.10/382.30±49.30	N/A	N/A	N/A	N/A	None.	Nausea, diarrhea: 18; liver damaged:6; icterus: 1.
Ying Lu, 2008 [28]	60/52/0/0	36/54/0/6	N/A	N/A	N/A	N/A	N/A	N/A	N/A	N/A
Jin Yang, 2010 [29]	8/12/7/3	10/12/6/2	N/A	N/A	N/A	N/A	N/A	N/A	None.	Nausea, diarrhea:4; hemameba reduced: 2.
Bing Ji, 2010 [30]	26/0/4/0	27/0/3/0	526.76±84.40/N/A	538.36±73.92/N/A	N/A	N/A	32.03±8.18/N/A	28.37±10.53/N/A	None.	11
Xiaoxia Wang, 2010 [31]	15/8/5/2	16/7/6/1	N/A	N/A	N/A	N/A	N/A	N/A	N/A	N/A
Yu Ding, 2011 [32]	10/18/10/2	4/12/12/8	N/A	N/A	N/A	N/A	N/A	N/A	N/A	N/A
Xizhou Zhang, 2011 [33]	8/16/15/6	3/14/14/14	556.37±87.82/406.54±51.07	549.56±79.87/426.23±67.65	N/A	N/A	N/A	N/A	None.	Nausea, diarrhea: 3; liver damaged:1; icterus: 1.
Mengyuan Liu, 2011 [34]	21/4/3/2	20/3/4/3	493.6±84.1/309.3±65.1	505.4±114.6/320.7±60.0	13.3±4.1/5.3±2.5	12.8±3.9/7.2±4.2	44.6±1.4/16.7±7.2	43.1±12.2/18.2±7.9	N/A	N/A
Yongkai Huang, 2011 [35]	8/6/0/1	8/5/0/2	480.88±110.54/390.67±98.23	476.68±109.76/490.88±112.54	35.24±20.15/9.01±2.02	37.89±21.69/8.97±1.98	30.09±19.90/14.98±7.92	31.67±20.76/15.12±8.15	N/A	N/A
Yadong Han, 2011 [36]	34/26/0/0	18/27/0/3	N/A	N/A	N/A	N/A	N/A	N/A	N/A	N/A
Baiyan Qin, 2011 [37]	29/10/6/0	17/13/10/6	601.20±133.5/367.1±97.3	598.6±129.7/417.7±89.2	25.11±9.21/11.14±5.43	24.67±9.54/13.22±6.03	49.71±11.05/22.04±7.27	49.71±11.05/22.04±7.27	N/A	N/A

??? Cure/markedly effective/effective/ineffective;

▴ EG Experimental Group, CG Control Group;

▪ ESR Erythrocyte Sedimentation Rate; N/A not applicable;

N/A not applicable;

♦ (B/A) Before intervention/After intervention.

### Serum uric acid concentration (µmol/L)

**Figure 2:**
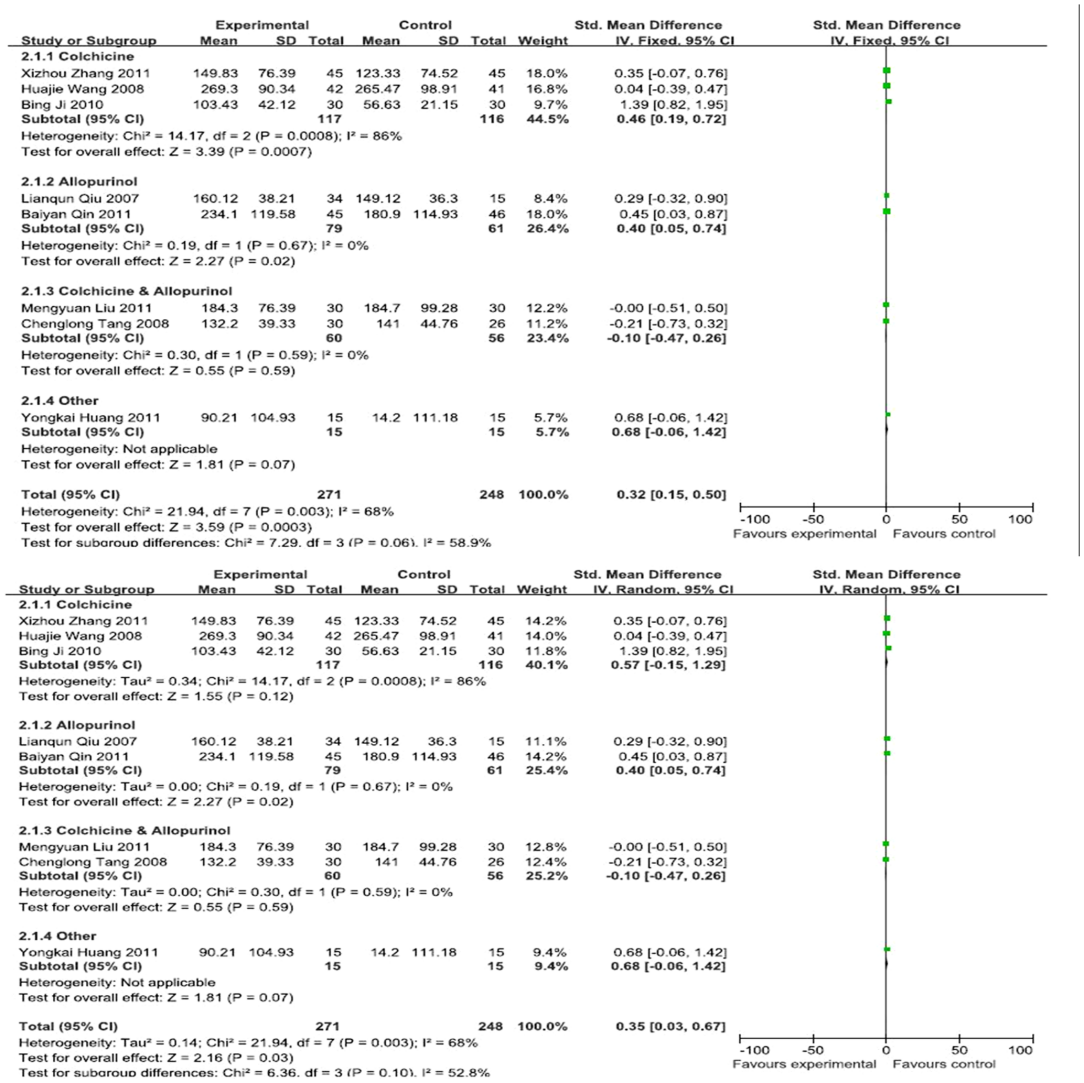
Effects of Chinese herbal decoction on serum uric acid in gout patients. ((1)Study of subgroup, first author and publish year; (2)Subgroups divided by western medicine ingredients: Colchicine, the control group have only contained colchicine; Colchicine & Allopurinol, the control group both only contained colchicine and allopurinol; Allopurinol, the control group have only contained allopurinol; Other, Diclofenac Sodium Enteric-coated Tablets or Meloxicam Tablets; Experimental: the group of Chinese herbal decoction; Control: the group of western medicine. (3)I-squared and *P* is the criterion of heterogeneity test; ♦pooled relative risk; —▪—,relative risk and 95 confidence interval.)

Eight RCTs provided the serum uric acid concentration data; these studies included 519 patients (271 cases in the experimental group and 248 cases in the control group) [24], [25], [26], [29], [32], [33], [34], [36]. By the subgroup meta-analysis, the results show that there was only a statistically significant between Chinese herbal decoction and Allopurinol (SMD, 0.40 (95%CI, 0.05–0.74);. Totally, Figure 2 showed that I^2^ = 68% (*P* = 0.003); therefore, the analysis used a random effects model, and the combined SMD was 0.35 with a 95% CI of 0.03 to 0.67. Therefore, Chinese herbal decoction and Western medicine showed significant differences in their ability to reduce the Serum uric acid concentration in patients. (**Figure 2**).

### C-reactive protein (mg/L)

**Figure 3:**
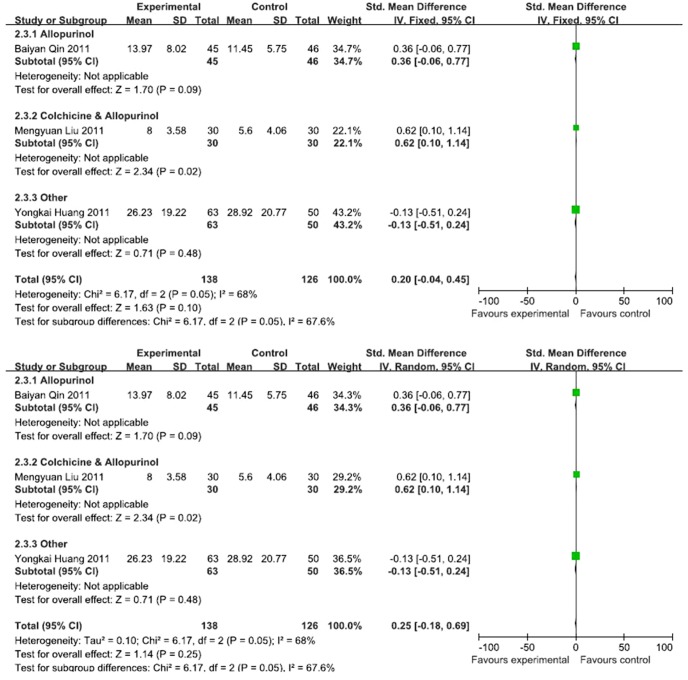
Effects of Chinese herbal decoction on C-reactive protein in gout patients. ((1)Study of subgroup, first author and publish year; (2)Subgroups divided by western medicine ingredients: Colchicine, the control group have only contained colchicine; Colchicine & Allopurinol, the control group both only contained colchicine and allopurinol; Allopurinol, the control group have only contained allopurinol; Other, Diclofenac Sodium Enteric-coated Tablets or Meloxicam Tablets; Experimental: the group of Chinese herbal decoction; Control: the group of western medicine. (3)I-squared and *P* is the criterion of heterogeneity test; ♦pooled relative risk; —▪—,relative risk and 95 confidence interval.)

Three RCTs provided the C-reactive protein data, and these studies included 181 patients (90 patients in the experimental group and 91 cases in the control group) [34], [35], [37]. And they should be divided into three subgroups, so it's meaningless for the subgroup meta-analysis. Totally, Figure 3 shows that I^2^ = 68%. A fixed effect model was adopted; the combined SMD was 0.25, and the 95% CI was −0.18 to 0.69. Therefore, significant differences were not found between the ability of the Chinese herbal decoction of the experimental group and the Western medicine of the control group to reduce C-reactive protein levels (**Figure 3**).

### Erythrocyte sedimentation rate (ESR) (mm/h)

**Figure 4:**
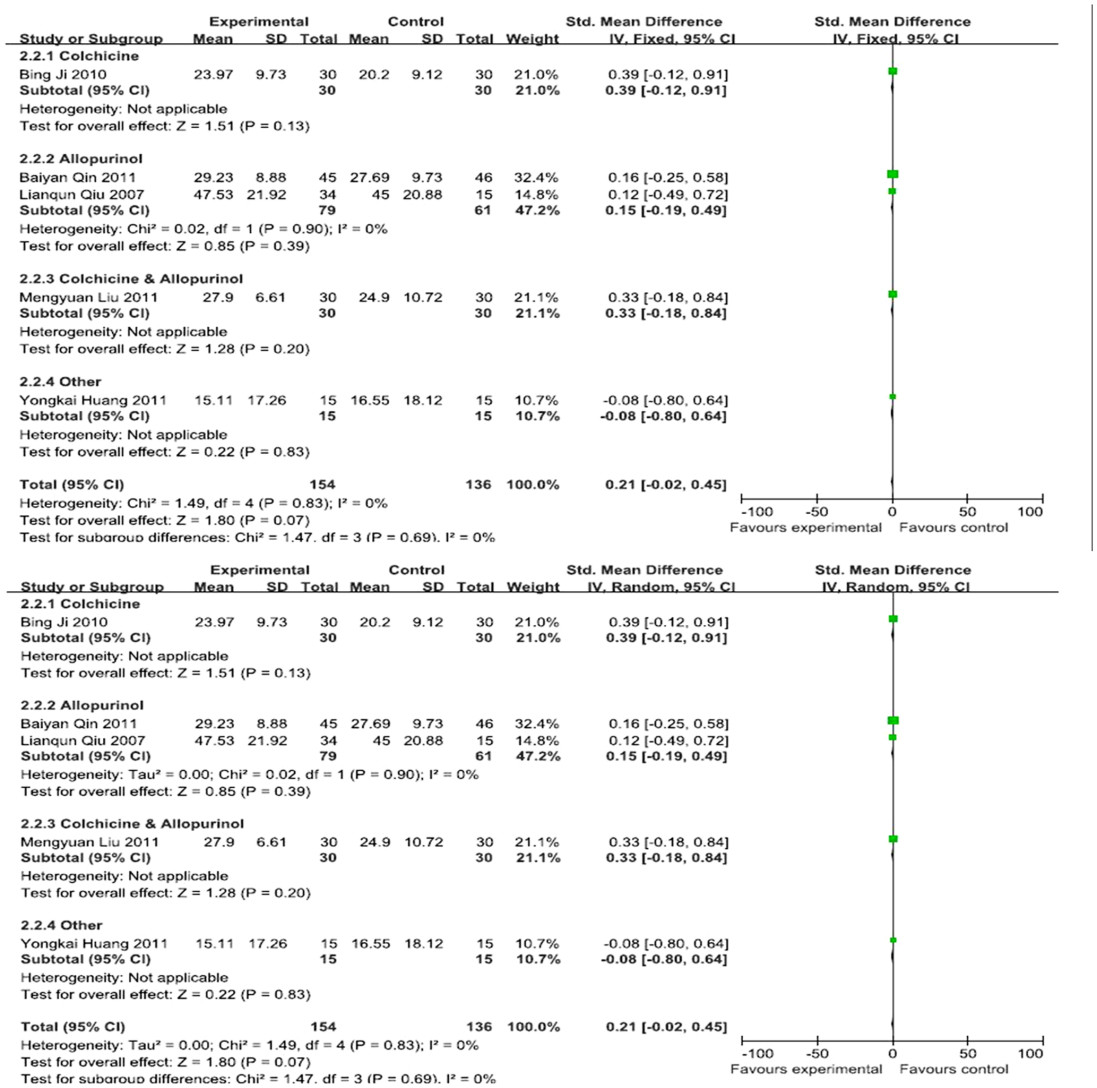
Effects of Chinese herbal decoction on ESR in patients with gout. ((1)Study of subgroup, first author and publish year; (2)Subgroups divided by western medicine ingredients: Colchicine, the control group have only contained colchicine; Colchicine & Allopurinol, the control group both only contained colchicine and allopurinol; Allopurinol, the control group have only contained allopurinol; Other, Diclofenac Sodium Enteric-coated Tablets or Meloxicam Tablets; Experimental: the group of Chinese herbal decoction; Control: the group of western medicine. (3)I-squared and *P* is the criterion of heterogeneity test; ♦pooled relative risk; —▪—,relative risk and 95 confidence interval.)

Five RCTs provided ESR data for 290 patients (154 cases in the experimental group and 136 cases in the control group) [25], [30], [34], [35], [37]. By the subgroup meta-analysis, there was no statistical significant between Chinese herbal decoction and 4 western medicine subgroups. As illustrated in Figure 4, I^2^ = 0%, and a fixed effects model was adopted for the analysis. The combined SMD was 0.21, and the 95% CI was −0.02 to 0.45. Therefore, there was no significant difference between the effects of the Chinese herbal decoction of the experimental group and the Western medicine of the control group on ESR (**Figure 4**).

### Overall efficacy of Chinese herbal decoction

**Figure 5:**
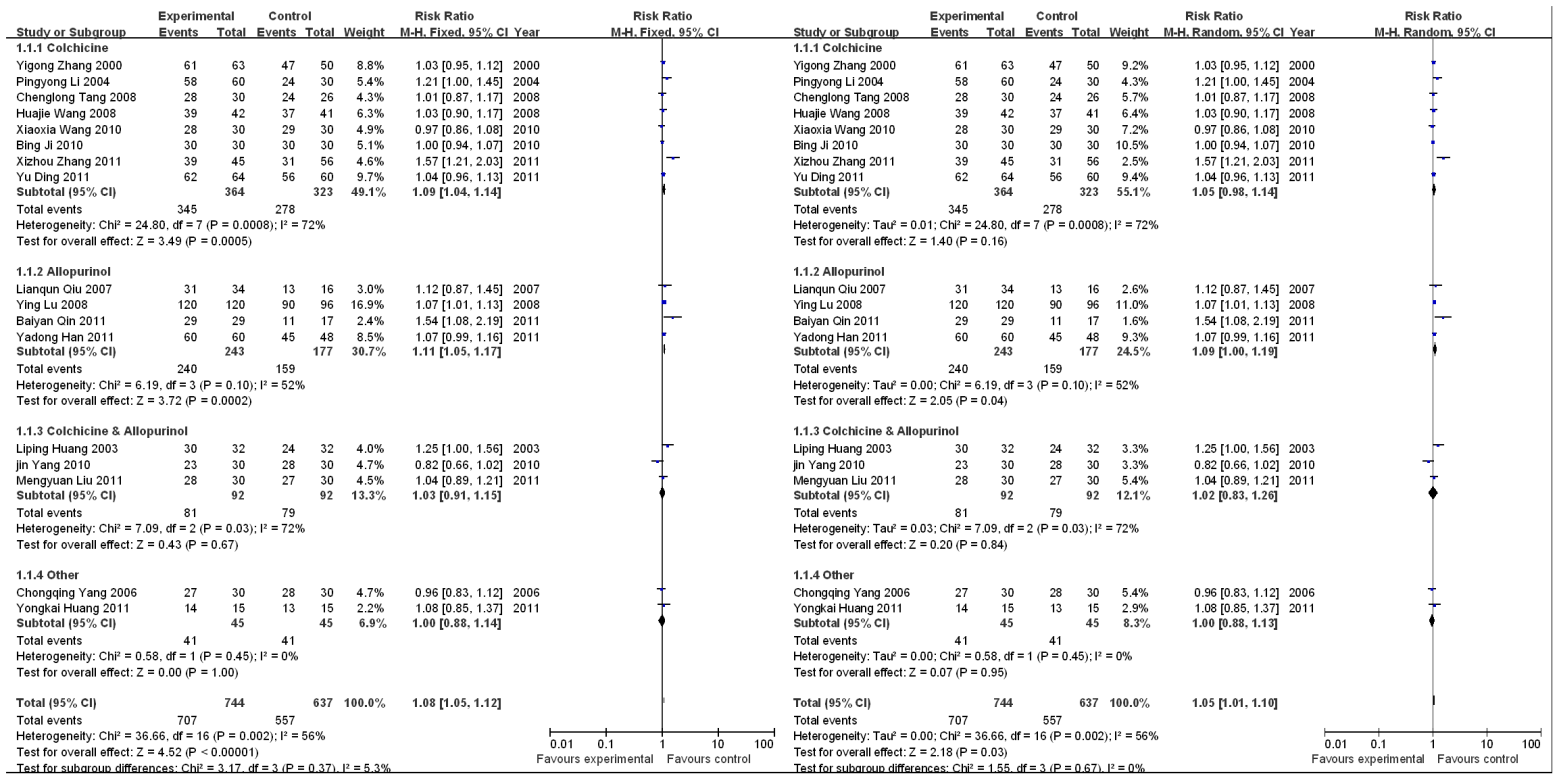
Analysis of the overall efficacy of Chinese herbal decoction and Western medicine for the treatment of gouty arthritis. ((1)Study of subgroup, first author and publish year; (2)Subgroups divided by western medicine ingredients: Colchicine, the control group have only contained colchicine; Colchicine & Allopurinol, the control group both only contained colchicine and allopurinol; Allopurinol, the control group have only contained allopurinol; Other, Diclofenac Sodium Enteric-coated Tablets or Meloxicam Tablets; Experimental: the group of Chinese herbal decoction; Control: the group of western medicine. (3)I-squared and *P* is the criterion of heterogeneity test; ♦ pooled relative risk; —▪—,relative risk and 95 confidence interval.)

Seventeen RCTs were analyzed, including 1,402 cases (737 cases in the experimental group and 665 cases in the control group). By the subgroup meta-analysis, there was no statistical significant between Chinese herbal decoction and 4 western medicine subgroups. Totally, the meta-analysis showed that I^2^ = 56%, and the analysis was performed using a Random effects model. The combined RR was 1.05, and the 95% CI was 1.01 to 1.10, indicating that Chinese herbal decoction and Western medicine exhibited a significant difference in their overall efficacy of treating gouty arthritis. (**Figure 5**).

### Adverse reactions

**Figure 6:**
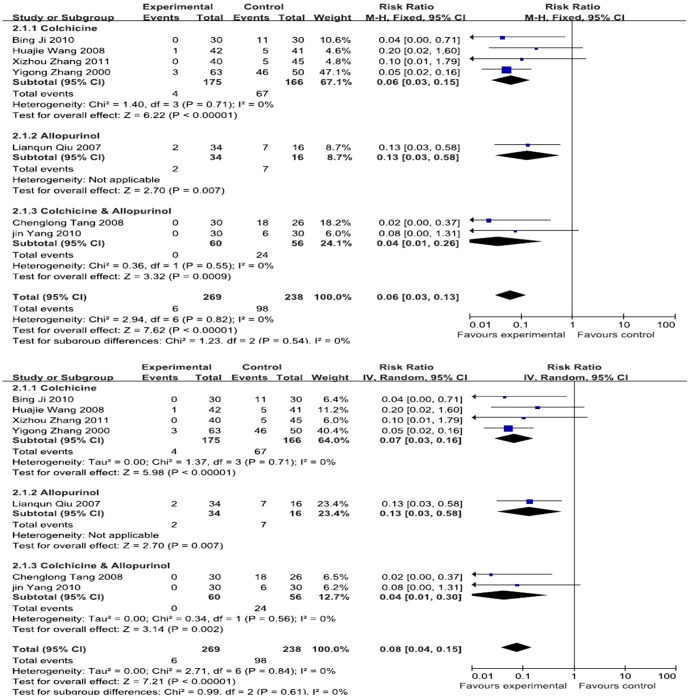
An analysis of the adverse reactions caused by Chinese herbal decoction and Western medicine in the treatment of gouty arthritis. ((1)Study of subgroup, first author and publish year; (2)Subgroups divided by western medicine ingredients: Colchicine, the control group have only contained colchicine; Colchicine & Allopurinol, the control group both only contained colchicine and allopurinol; Allopurinol, the control group have only contained allopurinol; Other, Diclofenac Sodium Enteric-coated Tablets or Meloxicam Tablets; Experimental: the group of Chinese herbal decoction; Control: the group of western medicine. (3)I-squared and *P* is the criterion of heterogeneity test; ♦ pooled relative risk; —▪—,relative risk and 95 confidence interval; ←, the trend pooled.)

Seven RCTs provided safety evaluation data, including 507 patients (269 cases in the experimental group and 238 cases in the control group) [21], [25], [26], [27], [29], [30], [33]. By the subgroup meta-analysis, there was statistical significant in all the subgroups (Colchicine, RR, 0.06 (95%CI, 0.03–0.13); Allopurinol 0.13(95CI, 0.03–0.58); Allopurinol & Colchicine, 0.04(95CI, 0.01–0.26)). Totally, the meta-analysis showed that I^2^ = 0%, and the analysis was performed using a fixed effects model. The combined RR value was 0.06, and the 95% CI was 0.03 to 0.13, indicating that there were overall fewer adverse reactions when using Chinese herbal decoction to treat gouty arthritis than when using Western medicine.(**Figure 6**)

### Sensitivity analysis

By model transformation between the fixed effects model and random effects model, the results of sensitivity analysis were stable. (**Figure 2** **–** **6**)

## Discussion

Due to the influence of such factors as diet, genetics, geographical region and mind, the incidence of gout has been gradually increasing worldwide every year [38], [39], [40]. Therefore, choosing a suitable clinical treatment approach is particularly important. According to this study, regarding treatment efficacy, Chinese herbal decoction and traditional Western medicine have a similar effect in reducing the blood uric acid and ESR in patients with gouty arthritis. Although the two treatments showed a statistically significant difference in reducing the C-reactive protein level of patients, the difference was not clinically significant. Similarly, although the two treatment methods showed a statistically significant difference (*P*<0.05) in the overall number of patient with effectively improved treatment outcomes, the difference in clinical efficacy was not significant (i.e., RR<1.1) [41], [42]. In summary, the clinical efficacies of the two treatment methods are the same. We believe that the main reason for this similarity is because the extracts of the Chinese herbal decoction ingredients contain colchicine and other alkaloids. The Chinese herbal decoction used by Chongqing Yang [24], Xiaoxia Wang [31] and others contains Pseudbubus Cremastra Seu pleiones. The bulbs of Pseudbubus Cremastra Seu pleiones contain the active ingredient colchicine [43]. Yu Ding [32] and Xizhou Zhang [33] and others used Lilium brownii var. viridulum instead of Pseudbubus Cremastra Seu pleiones, primarily because the chemical components of Lilium brownii var. viridulum include colchicine and other alkaloids [44]. In addition to Pseudbubus Cremastra Seu pleiones, the Chinese herbal decoctions used by Yongkai Huang [35], Bing Ji [30] and Baiyan Qin [37] also contained other herbs, such as Clematis chinensis Osbeck, Dioscorea collettii and Alisma orientale (Samuel), which provides additional functions including lowering blood pressure, promoting the excretion of uric acid, reducing blood sugar and reducing blood lipids [45]. Yadong Han [36], Huajie Wang [26] and others believe that adding a certain amount of Plantago to prescriptions has a significant effect on facilitating urine production and reducing uric acid and that this herb can also increase the excretion of urea and chloride [46], leading to a more significant treatment effect. The above examples fully demonstrate that Chinese herbal decoction and traditional Western medicine have similar efficacies in treating gout.

In terms of adverse reactions, traditional Western medicines, such as colchicine, probenecid, allopurinol alcohol and non-steroidal anti-inflammatory drugs, can produce anti-inflammatory and analgesic effects by reducing uric acid deposition and the associated inflammatory response. The above medications do not affect the formation, dissolution and excretion of uric acid salts and thus do not reduce blood uric acid levels [47]. However, these medications can cause a variety of adverse reactions, specifically gastrointestinal reactions, including abdominal pain, diarrhea, vomiting and loss of appetite. In severe cases, bone marrow suppression and liver and kidney damage can occur [48]; these adverse reactions can affect patient compliance to a certain extent. In contrast, Chinese herbal decoctions usually contain several condition-improving drug ingredients that work synergistically. For example, Jin Yang [29], Huajie Wang [26] and others add Rhizoma Ligustici wallichii to the prescription. This addition functions to resist platelet aggregation, improve microcirculation, expand small arteries, protect the kidneys and increase the rate of glomerular filtration [49]. These benefits may be among the reasons that Chinese herbal decoctions can effectively reduce the adverse drug reactions when treating gout.

All the Chinese herbal decoctions in this study were classified the type of Invigorate the circulation of blood [50]. Numerous studies have shown that in addition to alkaloids, Chinese herbal decoction also contains condition-improving drug ingredients. These condition-improving ingredients not only ease redness, swelling, heat, pain and other inflammatory response during the treatment of the acute arthritis phase of gout, but these ingredients also condition the visceral tissues to reduce adverse drug reactions while ensuring the therapeutic effect. However, the molecular biological mechanism through which Chinese herbal decoctions reduce or eliminate the adverse reactions caused by alkaloids remains unclear and need to be validated with additional studies.

**Figure 7:**
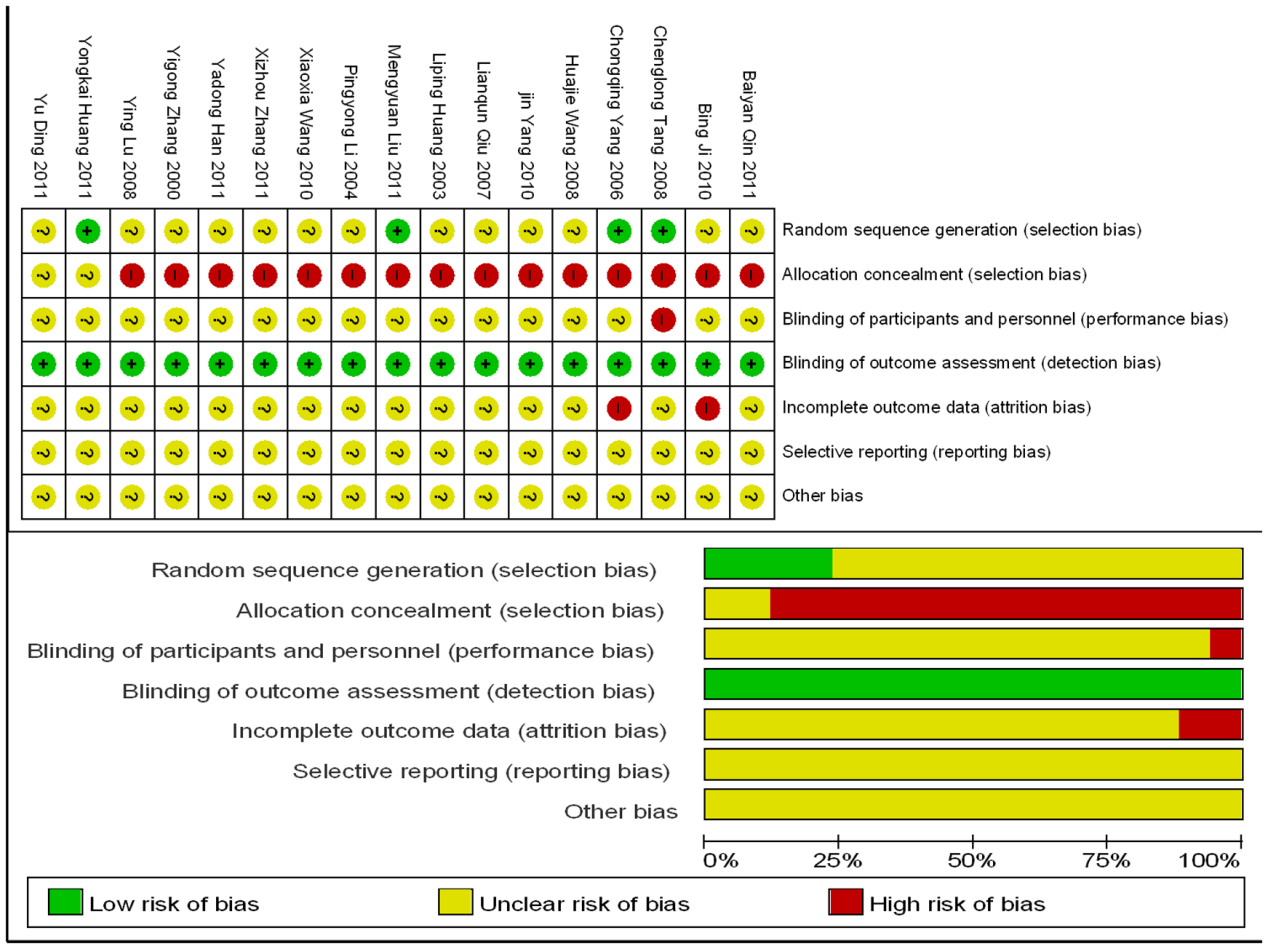
Risk of bias summary & Risk of bias graph.

The systematic review in our study has its own limitations, primarily due to the lack of high-quality RCTs. Most of the included RCTs are of low quality; high-quality RCTs only counted for 23.53% (4/17) of the studies. All of the RCTs utilized a random and blinded design, but only 23.53% (4/17) had a double-blind design and described the random design. The 88.24% (15/17) RCTs failed to mention concealed random allocation and the number of patients who quit the study or were lost during follow-up (**Figure 7**). In terms of adverse reactions, we only analyzed the statistical data of patients with liver and kidney dysfunction and gastrointestinal reactions, due to the lack of descriptions of the measurement data. Therefore, a more rational approach is necessary to evaluate the therapeutic effects of Chinese herbal decoction through the further development of multi-center and large-scale RCTs and to improve investigations into the adverse drug reactions of Chinese herbal decoction, thereby contributing to the body of knowledge concerning clinical evidence-based medical research.

Through a systematic review of the clinical efficacy and safety of Chinese herbal decoctions and traditional Western medicine for the treatment of gout, we found that Chinese herbal decoction and traditional Western medicine led to similar clinical efficacy, but the Chinese herbal decoctions were superior to Western medicine in terms of controlling adverse drug reactions. However, the pharmaceutical ingredients of Chinese herbal decoction are more complex; due to the lack of unified medication standards, pharmaceutical ingredients with similar efficacy are frequently interchanged in different prescriptions. In addition, the pharmacokinetics and molecular biological mechanism by which Chinese herbal decoction can treat patients with gout while producing reduced adverse drug reactions is unclear and requires further extensive and in-depth investigation.

## Supporting Information

Checklist S1 (DOC)
