# Supplementary material for: Systematic Review and Meta-Analysis of the Clinical Efficacy and Adverse Effects of Chinese Herbal Decoction for the Treatment of Gout
Source: PLoS One. 2014 Jan 21;9(1):e85008. doi: 10.1371/journal.pone.0085008 (PMC3897404; doi:10.1371/journal.pone.0085008)
Supplement: Checklist S1 — (DOC) [file pone.0085008.s001.doc]

PRISMA Checklist

| Section/topic | # | Checklist item | Reported on page # |
| --- | --- | --- | --- |
| TITLE | | | |
| Title | 1 | Systematic review and meta-Analysis of the clinical efficacy and adverse effects of Chinese herbal decoction for the treatment of gout | Title |
| ABSTRACT | | | |
| Structured summary | 2 | Background: Gout is an inflammatory response in the tissues against a foreign body and occurs when uric acid is deposited in the joints as a sodium salt because of abnormal purine metabolism. In East Asia, numerous reports describe the utilization of traditional Chinese herbal decoctions to treat gout. However, the reported clinical effects vary.  Objectives: In this study, we reviewed and analyzed a large number of randomized controlled clinical trials to systematically assess the clinical efficacy and adverse reactions of Chinese herbal decoctions for treating gout.  Methods: We performed a comprehensive search of databases, such as PubMed, EMBASE, the Cochrane Central Register of Controlled Trials, Chinese biomedical literature database, China Doctoral Dissertations Full-text Database, Chinese Scientific and Technological Journals Database and Traditional Chinese Medicine Database. In addition, we manually searched the relevant meeting information in the library of the Third Military Medical University.  Results: Finally, 17 randomized controlled trials with a sample size of 1,402 cases met the criteria and were included in the study. The results of the meta-analysis showed that when gout had progressed to the stage of acute arthritis, there was no significant difference in clinical efficacy between Chinese herbal decoctions and traditional Western medicine, as indicated based on the following parameters: serum uric acid (standardized mean difference (SMD): -0.27, 95% confidence interval (CI): -0.56 to 0.03), C reactive protein (SMD: -0.36, 95% CI: -0.65 to -0.06), erythrocyte sedimentation rate (SMD: -0.16, 95% CI: -0.40 to 0.07) and overall clinical response (relative risk (RR): 1.08, 95% CI: 1.04 to 1.11). However, the Chinese herbal decoction was significantly better than traditional Western medicine in controlling adverse drug reactions (RR: 0.06, 95% CI: 0.03 to 0.13).  Condusions: Through a systematic review of the clinical efficacy and safety of Chinese herbal decoctions and traditional Western medicine for the treatment of gout, we found that Chinese herbal decoction and traditional Western medicine led to similar clinical efficacy, but the Chinese herbal decoctions were superior to Western medicine in terms of controlling adverse drug reactions. However, the pharmaceutical ingredients of Chinese herbal decoction are more complex; due to the lack of unified medication standards, pharmaceutical ingredients with similar efficacy are frequently interchanged in different prescriptions. In addition, the pharmacokinetics and molecular biological mechanism by which Chinese herbal decoction can treat patients with gout while producing reduced adverse drug reactions is unclear and requires further extensive and in-depth investigation. | Abstract |
| INTRODUCTION | | | |
| Rationale | 3 | The clinical designs in all reports included in this study were clinical randomized controlled trials (RCTs). The trials were divided into experimental and control groups based on the intervention methods, with the experimental group receiving the Chinese herbal decoction alone and the control group receiving only Western medicine. The time of publication was restricted to the period from January 2001 to June 2012, and the journals’ languages were restricted to Chinese and English. | Materials and Methods |
| Objectives | 4 | Participants：According to the diagnostic criteria created by the American Rheumatism Association in 1977, all subjects included in the study were diagnosed with primary gout in the phase of acute arthritis. Subjects with secondary gout due to blood diseases, kidney disease or cancer were excluded. Pregnant women and patients with serious heart, liver or kidney disorders or other primary diseases were also excluded.  Interventions： The major treatments of gout used traditional Chinese herbal decoction were included, rather than other Chinese patent medicines (e.g., particle, tablets and capsules, etc.). And some other non-oral treatments like acupuncture, qigong and emplastrums were excluded. Comparisons：Study subjects were divided into test group and control group followed by RCT. Traditional Chinese herbal decoction were used in test group, only oral prescription medicine treatments were included. In control group, normal western medicine were used to treat gout patients in the course of 15 ~30 days, such as colchicine and allopurinol non-steroid medicine. And then, the study discussed a comparison of follow-up adverse effects between test group and control group.  Outcomes： Enumeration data were divided into 4 categories of cure, markedly effective, effective and ineffective in accordance with the standards of “Guiding Principles for the Clinical Investigation of New Traditional Chinese Medicine” . The number of patients with effective treatment results in each group was counted based on the categories of cured, markedly effective and effective. Measurement data included three primary indicators: blood uric acid, C-reactive protein and erythrocyte sedimentation rate (ESR).  study design： A randomized controlled trial meta - analysis | Materials and Methods |
| METHODS | | | |
| Protocol and registration | 5 | No. |  |
| Eligibility criteria | 6 | The literature inclusion criteria were as follows: (1) According to the diagnostic criteria created by the American Rheumatism Association in 1977, all subjects included in the study were diagnosed with primary gout in the phase of acute arthritis. Subjects with secondary gout due to blood diseases, kidney disease or cancer were excluded. Pregnant women and patients with serious heart, liver or kidney disorders or other primary diseases were also excluded; (2) The clinical designs in all reports included in this study were clinical randomized controlled trials (RCTs), The trials were divided into experimental and control groups based on the intervention methods, with the experimental group receiving the Chinese herbal decoction alone and the control group receiving only Western medicine; (3) The analysis of balance between the groups showed that the baseline data were comparable; (4) Endpoint indicators: Guiding Principles for the Clinical Investigation of New Traditional Chinese Medicine; blood uric acid, C-reactive protein and erythrocyte sedimentation rate (ESR). | Materials and Methods |
| Information sources | 7 | PubMed, Medline, Chinese Biomedical Literature Database, China Doctoral Dissertations Full-text Database, Chinese Scientific and Technological Journals Database, Traditional Chinese Medicine Database, China Doctoral Dissertations Full-text Database, China Master Dissertations Full-text Database and CENTRAL from the Cochrane Library during the period from January 2001 to June 2012.And the library of the Third Military Medical University. | Materials and Methods |
| Search | 8 | #1 gout or podagra  #2 hyperuricemia  #3 Chinese medicine or Chinese herbal medicine or traditional Chinese medicine  #4 #1 and #2 and #3( Please see Manuscript Page 6 Line 5-8 ) | Materials and Methods |
| Study selection | 9 | A total of 786 research articles on the treatment of gout using traditional Chinese medicine were identified by searching the electronic databases. Based on the inclusion and exclusion criteria, 377 articles, including duplicated publications, articles with mismatched titles and articles with mismatched subjects, were excluded. The remaining 324 articles were reviewed thoroughly; 95 non-RCT articles, 66 articles based on animal experiments, 178 articles in which the medicines used for the experimental group or the control group did not comply with the inclusion criteria, 6 articles in which patients with gout that had not progressed into gouty arthritis and 15 articles in which patients had secondary gout or had other complications were excluded. Thus, 17 studies were included in this study for the systematic review. | Results |
| Data collection process | 10 | Three investigators participated in the data extraction of all publications included in the study. Information, including the first author, publication year, total number of cases included in the experimental group and the control group, intervention methods and endpoint evaluation indicators, was extracted. One investigator (XL) first performed the data extraction, and the second investigator (LL2) subsequently re-examined the publication and verified the results. Differences were discussed with the third investigator (PC), and consensus was reached by discussion. | Materials and Methods |
| Data items | 11 | Table 1. Characteristics of the included studies: author/year, sample size, age, disease severity, intervention methods, duration treatment; Table 2. Outcomes: author/year, the overall efficacy, Blood uric acid concentration (μmol/L), C-reactive protein (mg/L), ESR (mm/h), adverse reactions. | Results |
| Risk of bias in individual  studies | 12 | The systematic review in our study has its own limitations, primarily due to the lack of high-quality RCTs. Most of the included RCTs are of low quality; high-quality RCTs only counted for 23.53% (4/17) of the studies. All of the RCTs utilized a random and blinded design, but only 23.53% (4/17) had a double-blind design and described the random design. The 88.24% (15/17) RCTs failed to mention concealed random allocation and the number of patients who quit the study or were lost during follow-up. | Discussion |
| Summary measures | 13 | The measurement data were combined using the standardized mean difference (SMD) and 95% confidence interval (CI), and the enumeration data were evaluated using the relative risk (RR) and 95% CI based on the number of patients with effective treatment results in the combined experimental group and control group. Stata11.0 software was used to analyze the collected clinical research data. | Materials and Methods |
| Synthesis of results | 14 | If the heterogeneity across the studies was within the acceptable range (I² < 50%), a fixed effects model was used to combine the studies. Otherwise, a random effects model was used. | Materials and Methods |
| Risk of bias across studies | 15 | The quality of the articles included in this study was assessed using the Cochrane Handbook for Systematic Reviews of Interventions and Jadad scoring [18,19]. Two reviewers (XL and LL2) performed blinded independent evaluation. If inconsistent evaluation results were obtained, a third reviewer (LZ) intervened, and consensus was reached by discussion. The details that were assessed were as follows: 1) whether the test methods were random, 2) whether allocation concealment was achieved, 3) whether blinded tests were adopted and 4) whether patients were lost due to follow-up or quit. The scoring scale was 1-7 (1-3 indicated low quality, and 4-7 indicated high quality). | Discussion |
| Additional analyses | 16 | Sensitivity analyses & subgroup-meta-analysis | Results |
| RESULTS | | | |
| Study selection | 17 | A total of 786 research articles on the treatment of gout using traditional Chinese medicine were identified by searching the electronic databases. Based on the inclusion and exclusion criteria, 377 articles, including duplicated publications, articles with mismatched titles and articles with mismatched subjects, were excluded. The remaining 324 articles were reviewed thoroughly; 95 non-RCT articles, 66 articles based on animal experiments, 178 articles in which the medicines used for the experimental group or the control group did not comply with the inclusion criteria, 6 articles in which patients with gout that had not progressed into gouty arthritis and 15 articles in which patients had secondary gout or had other complications were excluded. Thus, 17 studies were included in this study for the systematic review. | Results |
| Study characteristics | 18 | Please see Table 1. | Results |
| Risk of bias within studies | 19 | The systematic review in our study has its own limitations, primarily due to the lack of high-quality RCTs. Most of the included RCTs are of low quality; high-quality RCTs only counted for 23.53% (4/17) of the studies. All of the RCTs utilized a random and blinded design, but only 23.53% (4/17) had a double-blind design and described the random design. | Discussion |
| Results of individual studies | 20 | Please see Table 2. | Results |
| Synthesis of results | 21 | Please see Figure 2-6. | Results |
| Risk of bias across study | 22 | The systematic review in our study has its own limitations, primarily due to the lack of high-quality RCTs. Most of the included RCTs are of low quality; high-quality RCTs only counted for 23.53% (4/17) of the studies. All of the RCTs utilized a random and blinded design, but only 23.53% (4/17) had a double-blind design and described the random design. | Discussion |
| Additional analysis | 23 | Sensitivity analyses | Results |
| DISCUSSION | | | |
| Summary of evidence | 24 | Numerous studies have shown that in addition to alkaloids, Chinese herbal decoction also contains condition-improving drug ingredients. These condition-improving ingredients not only ease redness, swelling, heat, pain and other inflammatory response during the treatment of the acute arthritis phase of gout, but these ingredients also condition the visceral tissues to reduce adverse drug reactions while ensuring the therapeutic effect. However, the molecular biological mechanism through which Chinese herbal decoctions reduce or eliminate the adverse reactions caused by alkaloids remains unclear and need to be validated with additional studies. | Discussion |
| Limitations | 25 | Risk of bias: see item 12 & 15;  Incomplete retrieval of identified research: Delimitation of inclusion, e.g., restricts included papers to Chinese or English.  Reporting bias: Not found. | Discussion |
| Conclusions | 26 | Through a systematic review of the clinical efficacy and safety of Chinese herbal decoctions and traditional Western medicine for the treatment of gout, we found that Chinese herbal decoction and traditional Western medicine led to similar clinical efficacy, but the Chinese herbal decoctions were superior to Western medicine in terms of controlling adverse drug reactions. However, the pharmaceutical ingredients of Chinese herbal decoction are more complex; due to the lack of unified medication standards, pharmaceutical ingredients with similar efficacy are frequently interchanged in different prescriptions. In addition, the pharmacokinetics and molecular biological mechanism by which Chinese herbal decoction can treat patients with gout while producing reduced adverse drug reactions is unclear and requires further extensive and in-depth investigation. | Discussion |
| FUNDING | | | |
| Funding | 27 | The study was supported by three Natural Science Foundation of China (No.81172773, No.30872184 and No.30901242). | We have removed the funding from the manuscript. |
